# MicroRNA-134 regulates poliovirus replication by IRES targeting

**DOI:** 10.1038/s41598-017-12860-z

**Published:** 2017-10-04

**Authors:** Abhijeet A. Bakre, Byoung-Shik Shim, Ralph A. Tripp

**Affiliations:** 1 College of Veterinary Medicine, Department of Infectious Diseases, University of Georgia, Athens, 30602 GA USA; 2 Present Address: Department of Immunology and Microbial Science, The Scripps Research Institute, Jupiter, 33458 Florida USA

## Abstract

Global poliovirus eradication efforts include high vaccination coverage with live oral polio vaccine (OPV), surveillance for acute flaccid paralysis, and OPV “mop-up” campaigns. An important objective involves host-directed strategies to reduce PV replication to diminish viral shedding in OPV recipients. In this study, we show that microRNA-134-5p (miR-134) can regulate Sabin-1 replication but not Sabin-2 or Sabin-3 via direct interaction with the PV 5′UTR. Hypochromicity data showed miR-134 binding to Sabin-1 and 3 but not Sabin-2 IRES. Transfection of a miR-134 mimic repressed translation of Sabin-1 5′UTR driven luciferase validating the mechanism of miR-134-mediated repression of Sabin-1. Further, site directed mutagenesis of the miR-134 binding site in Sabin-1 IRES relieved miR-134-mediated repression indicating that these regulatory molecules have an important role in regulating the host gene response to PV. Binding of miR-134 to Sabin-1 IRES caused degradation of the IRES transcript in a miR-134 and sequence specific manner. The miR-134 binding site was found to be highly conserved in wild type PV-1 as well as EV71 strains indicating that miR-134 may regulate function of these IRES sequences in circulation.

## Introduction

Poliovirus (PV), a member of the *Picornaviridae* family, is a non-enveloped positive-sense single-strand RNA virus and the cause of poliomyelitis^1^. PV is transmitted by person-to-person contact, contamination, or by fomites^2^. Infection can be abortive, mild, or cause paralytic forms of illness. Abortive infections are typically symptom-free, mild infections (~24%) are associated with influenza-like symptoms, while paralytic infections (<1%) affect the neurons and can lead to a level of paralysis, disability, or death^3,4^. While there are no antiviral drugs approved for treatment of PV, vaccination has reduced the health burden by >99% worldwide^5^. Two major types of PV vaccines are currently in use worldwide - live-attenuated oral polio vaccine (OPV) and inactivated polio vaccine (IPV). Vaccinees today receive a mixture of genotypes (Sabin-1, Sabin-3). Though OPV and IPV confer lifelong protection, OPV vaccinees can shed and contribute to vaccine-derived PV (VDPV), immune deficiency-associated PV (iVDPV) or circulating vaccine-derived poliovirus (cVDPV) causing concerns of reversion to wild type. Thus, one must move away from OPV to IPV to stop PV spread. Additionally, as recommended by the National Research Council, there is a sustained effort to develop anti-PV therapeutics to target immune deficient PV reservoirs and shedders, and several agents that show anti-PV activity are being investigated^6–8^.

PV is generally acquired through the oral route and infects the gut epithelium via binding to the PV receptor (CD155)^9^. Infection of the gut epithelium is followed by rapid shutdown of host translational machinery^10^, unlinking of the VPg that enables the positive-strand RNA genome to function as a mRNA^11^ and VPg-mediated initiation of PV negative and positive-strand synthesis^12^. PV polyprotein translation critically depends on an internal ribosomal entry site (IRES) in the 5′UTR which is highly structured and binds to multiple host translational initiation factors. Mechanisms of how PV regulates host translation are well-characterized^13^, however the roles of host cellular non-coding RNAs in PV replication remain poorly understood and discovery can lead to the development of novel intervention strategies. Small non-coding regulatory RNAs (sncRNAs) are central to gene regulation with short interfering RNAs (siRNAs) and miRNAs being the two major means that regulate endogenous post-transcriptional gene expression^14,15^. miRNAs encoded in the genome can catalyze degradation of target transcripts or block translation by a short complementarity match between the miRNA “seed” site and a miRNA recognition element (MRE) in the 3′untranslated region (UTR) of a mRNA^16^. Computational and experimental evidence suggest that up to 60% of the eukaryotic transcriptome is under miRNA regulation^17^, and thus this class of regulatory molecules has important roles in determining host gene response to infection as well^16^.

Previous genome-wide PV screens^18^ have helped identify host genes that modulate PV replication, and data suggest that modulation of host mRNAs and miRNAs can be used to alter viral replication^19–21^. Genome-wide screens to determine pro-PV or anti-PV host genes have enabled development of enhanced vaccine cell lines^22^ to increase vaccine production and aid eradication. Similarly, studies have shown that replication of viruses carrying microRNA response elements (MREs) are abrogated in tissues expressing the cognate miRNA and may aid vaccine development^23–27^. We previously showed that PV replication is modulated by alterations in miR-555 activity^28^. Increasing miR-555 activity inhibits a host factor critical for PV replication, heterogeneous nuclear ribonucleoprotein C1/C2 (hnRNP C)^29^, which inhibits PV replication^28^.

Notably, we previously showed that miR-134 regulated host cell pathways involving Ras-related nuclear protein (RAN) which can control PV replication in a strain and cell type-independent manner^30^. However, post-infection upregulation of miR-134 function inhibited only Sabin-1, but not Sabin-2 replication. In this study, we show that miR-134 can directly target PV replication by targeting IRES mediated translation in Sabin-1, but not Sabin-2. We demonstrate that this strain-specific inhibition is mediated by complementarity between the miR-134 seed site, and the domain 5 of Sabin-1 IRES which inhibits initiation of viral translation. Additionally, we show that miR-134 binding to Sabin-1 UTR causes degradation of the IRES region. Further, sequence analysis of Sabin-1 and EV71 sequences suggests that the miR-134 binding site is highly conserved in circulating PV-1 and EV71 strains but is absent in PV-2 (Sabin-2) strains. These findings are important as they provide a better understanding of virus-host interaction and potentially a solution for therapeutic outcomes.

## Results

### MiR-134 represses Sabin-1 but not Sabin-2 or Sabin-3 replication

**Figure 1:**
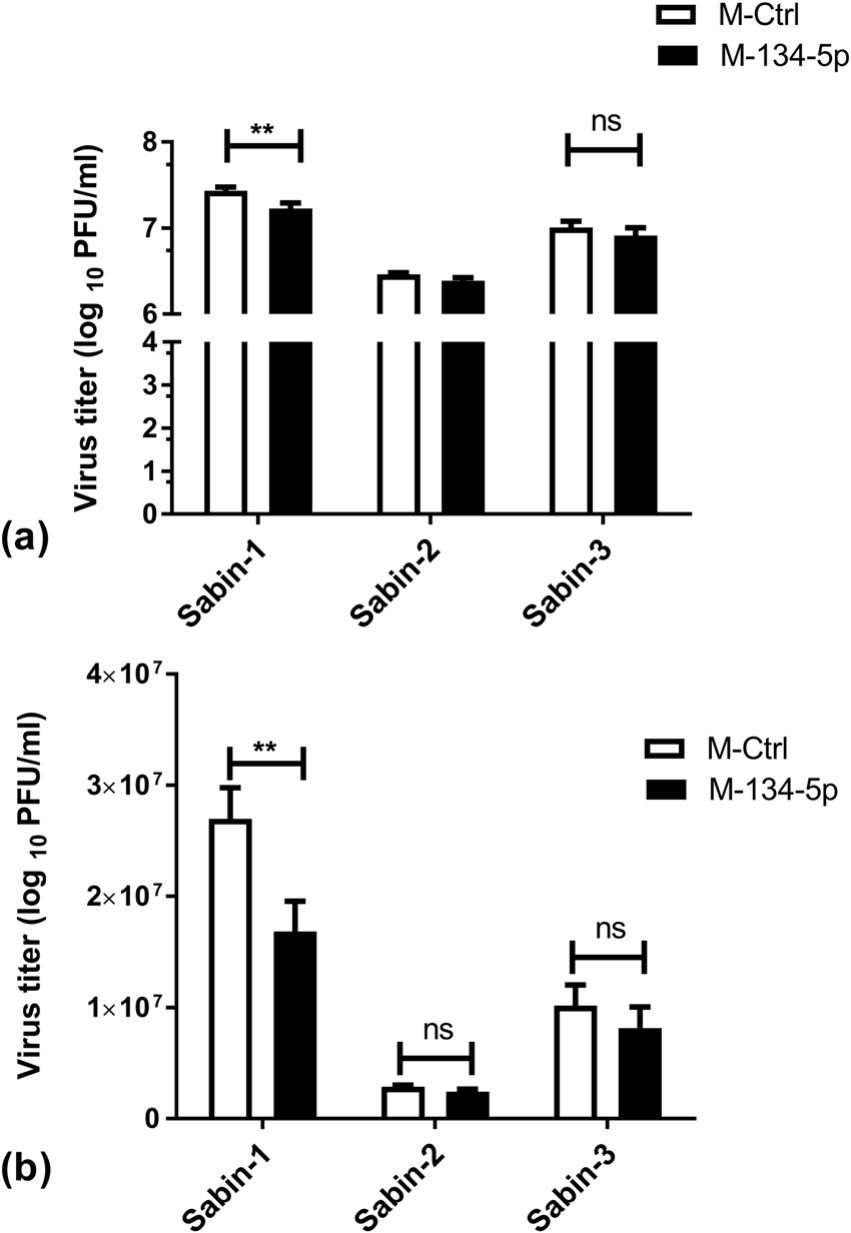
Sabin-1 replication is inhibited by miR-134 post-infection. (**a**) HEp-2 cells were infected with Sabin-1, Sabin-2, or Sabin-3 and transfected with control or a miR-134 mimic (25 nM). Cell supernatants were assayed for viral titers on HEp-2 cells. Error bars represent ± SEM from two independent experiments with triplicate replicates each. *p < 0.05, **p < 0.01, ***p < 0.001 ****p < 0.0001. Viral titers are shown as log _10_ pfu/ml. (**b**) Data in (**a**) are shown on a linear scale.

We recently showed that upregulation of miR-134 during PV infection causes a reduction in replication for both Sabin-1, Sabin-2 and Sabin-3, as well as Enterovirus 71 (EV71)^30,31^. We demonstrated that miR-134 downregulation of Ras-related nuclear protein (RAN) inhibits both PV and EV71 replication. EV71 repressed miR-197 causes RAN upregulation leading to increased nuclear transport of viral protein 3D/3CD and hnRNPK supporting increased viral replication^30,32^; increasing miR-134 led to reduced RAN and repressed PV replication. Upregulation of miR-134 using a miRNA mimic inhibited RAN and siRNA-mediated RAN silencing reduced Sabin-2 replication. In contrast replication of all three PV viruses in miR-134 inhibitor transfected cells was comparable to mimic negative control ﻿(MNC1) supporting our hypothesis that miR-134 upregulation abrogated viral replication. While expecting opposite phenotypes with mimic vs inhibitor transfections would be ideal, it is not always observed for multiple reasons (miRNA expression levels, cell cycle state, stability of the miRNA:inhibitor duplex etc). miRNAs can regulate various co-expressed genes due to the complementarity with their target MREs^17^. Commercial miRNA mimics are designed to resist intra-cellular degradation and are stable for long periods. Thus, typical transfections with miRNA mimics can significantly knock down expression of multiple target gene(s)/pathways over the typical 48 h transfection cycle and exhibit off-target effects making it difficult to dissect miRNA regulated pathways that alter viral replication. MiRNAs can also directly inhibit viral replication^33–43^. Inhibition of host protein synthesis following PV infection is well-established^13^. To determine if a miR-134 mimic could target the PV viral genome, cells were infected with Sabin-1, Sabin-2 or Sabin-3 viruses (MOI = 0.01) for 2 h to allow attachment and entry, followed by miR-134 mimic transfection (25 nM). This short transfection period allows examination of the direct effects of miR-134 mimic on Sabin-1 vs Sabin-2/3 infection and is not expected to significantly alter the pathways that would be modulated otherwise. Viral titers were determined at 24 h pi by plaque assay^31^. Transfection with the miR-134 mimic significantly (p < 0.01) modified Sabin-1 replication but had no detectable impact on Sabin-2 or Sabin-3 replication (Fig. 1). These data suggest that miR-134 regulates replication of Sabin-1, but not Sabin-2 or Sabin-3, and suggests that miR-134 regulates host machinery necessary for Sabin-1 replication to proceed.

Since rapid inhibition of viral protein synthesis is needed for controlling PV replication, we hypothesized that miR-134 was differentially inhibiting internal ribosomal entry site (IRES)-mediated translation of Sabin-1 during early stages of PV replication. Analysis of genomic vRNA sequences of Sabin-1, Sabin-2 and Sabin-3 using ViTA (a method for prediction of host miRNA targets on viruses)^44^ identified multiple potential miR-134 binding sites in all the three PV strains with the most in Sabin-1 (Table 1). A majority of these binding sites were in the coding regions which are highly conserved across PV strains (Supplementary Fig. S1). Among all sites, binding in the PV IRES (nt 473–493) was predicted to be most stable (ΔG = −21.8) (Table 1) for Sabin-1. This 5′UTR region containing the IRES has been shown to be crucial for initial PV translation of the polyprotein^45,46^. Comparative studies between attenuated PV vaccine strains and PV progenitor strains that are neurovirulent have identified three major determinants of attenuation at nt 472^47^, 480^48^ and 481^49^ in this IRES^49^. Additionally, reversion of nt 472 to wild type in an attenuated Sabin-3 strain was shown to restore virulence^50^.

**Table 1:** Predicted binding sites of miR-134 in the PV genomes.

	Rank	Target Location in viral genome and encoding viral gene		Mean free energy of folding (MFE)	ViTA Score	# of base pairs complementary to miR-134-5p	
						Watson Crick	Non-Watson-Crick
Sabin-1	1	6223–6249	P3	−18.7	141	18	1
	2	473–493	5′IRES	−21.8	143	14	3
	3	3652–3672	2A	−18.9	145	14	3
	4	6845–6865	P3	−19.6	152	14	3
	5	1805–1830	VP3	−17.0	164	16	2
Sabin-2	1	6844–6864	P3	−18.1	149	14	3
	2	7069–7086	P3	−18.8	167	15	1
Sabin-3	1	5610–5632	3D	−18.6	145	15	1
	2	4772–4793	2C	−19.76	147	15	1
	3	7058–7078	P3	−16.6	163	14	1

**Figure 2:**
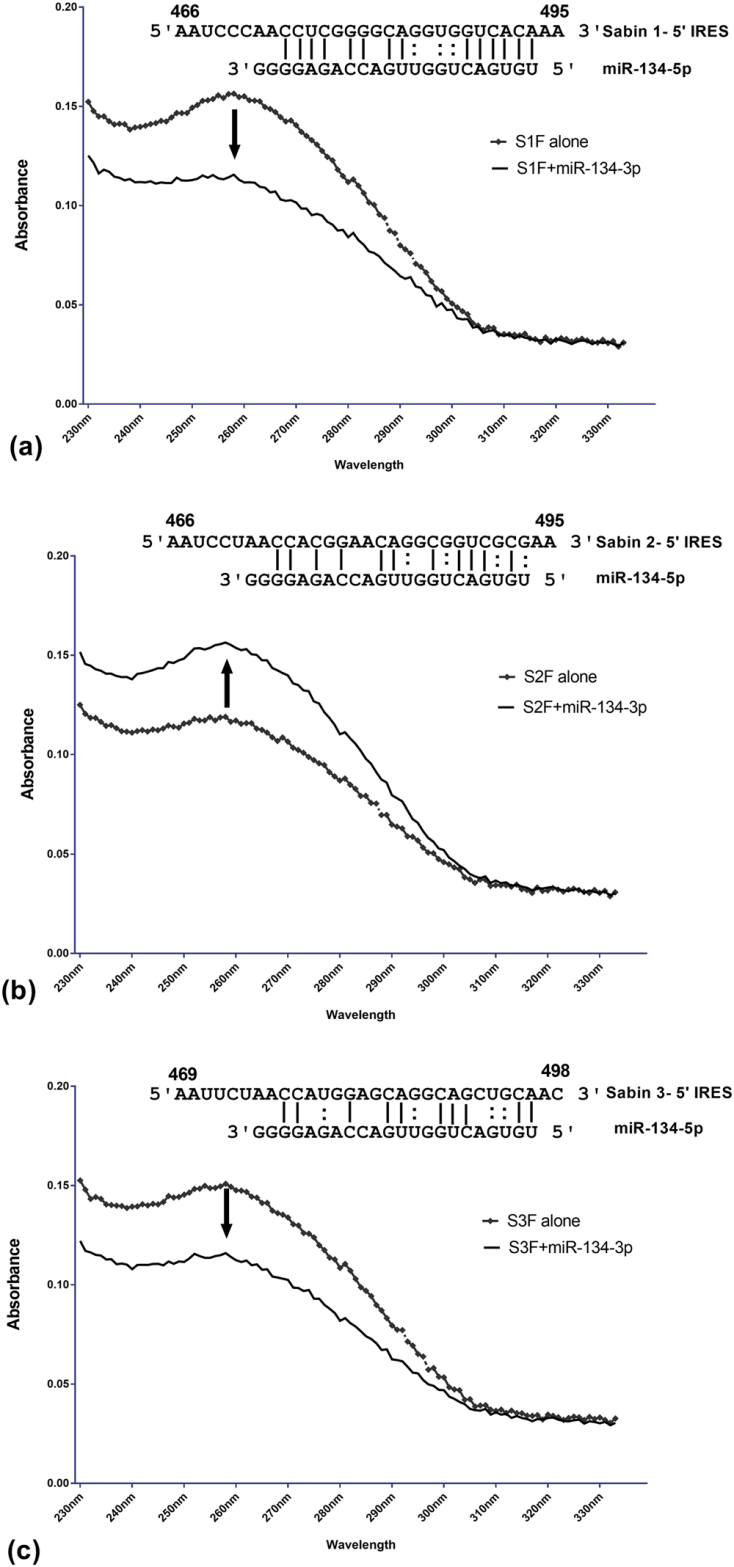
MiR-134 interacts with Sabin-1 and Sabin-3 but not Sabin-2. Absorbance profiles of renatured Sabin-1, Sabin-2, Sabin-3 oligonucleotides alone or with miR-134 were measured using UV spectrophotometry. Profiles represent hypochromic shifts in case of Sabin-1 (**a**) and Sabin-3 (**c**) and a hyperchromic shift for Sabin-2 (**b**). Data represent means of multiple measurements on six independent assays. Alignments show base pairing between viral regions and miR-134. Solid lines indicate Watson-Crick base pairs while colon symbols represent non-Watson-Crick pairs.

Domain V in the PV IRES (nt 472 onwards) showed distinct differences between Sabin-1, Sabin-2, and Sabin-3. Sabin-1 miR-134 binding prediction involved a contiguous stretch of six Watson-Crick (WC) base-pairs in the miRNA seed site followed by two wobble G:U pairs and was predicted to be stable (ΔG = −11.96) per the DINAmelt server^51^. In contrast, miR-134 binding for Sabin-2 and Sabin-3 was predicted to be less stable (ΔG = −7.3 for Sabin-2 and ΔG = −7.95 for Sabin-3) owing to lesser WC and non-WC base pairing in the seed site (Fig. 2a–c). Thus, Sabin-1:miR-134 sequences are predicted to rapidly anneal as shown by recent studies demonstrating that a 7 bp complementarity is critical for rapid RNA/DNA duplex formation^52^, and may be key to siRNA/miRNA “seed” based gene silencing in the canonical model. This 7-mer complementarity is also considered core to miRNA recognition of their target mRNAs^53^, thus these data suggest that the Sabin-1, but not Sabin-2 or Sabin-3 IRES sequences, are target for miR-134 regulation. Thus, we hypothesized that miR-134 would be able to form duplexes with Sabin-1, but not Sabin-2 and Sabin-3 IRES.

Inter-/intra-molecular base pairing in DNA/RNA molecules can appreciably reduce UV light absorbance leading to a hypochromic shift^54^. Conversely, non-complementary DNA and RNA strands increase absorbance of the solution and can lead to a hyperchromic UV shift due to additive absorption^55^. To determine if miR-134 interacted and hybridized to the IRES of Sabin-1, oligonucleotides containing these regions from Sabin-1, Sabin-2 and Sabin-3 were synthesized. We assumed that miR-134 and Sabin-1 would cause a “hypochromic”^54^ shift due to base-pairing. Heat-denatured solutions containing only Sabin-1, Sabin-2, and Sabin-3 or miR-134 oligonucleotides showed characteristic UV absorbance peaking at ~260 nm as expected (Fig. 2a–c). However, the addition of heat-dissociated miR-134 to Sabin-1, Sabin-2, or Sabin-3 solutions followed by renaturation at physiological temperatures produced a hypochromic shift in Sabin-1 (Fig. 2a) and Sabin-3 (Fig. 2c), but not for Sabin-2, suggesting that miR-134 can base pair with Sabin-1 and Sabin-3 but not Sabin-2 (Fig. 2a and b). Lack of binding between the Sabin-2 oligonucleotides and miR-134 produced a hyperchromic shift (Fig. 2b) as expected. This is also supported by the higher numbers of Watson-Crick base pairs between Sabin-1 and miR-134 relative to Sabin-2 and Sabin-3 (Table 1).

**Figure 3:**
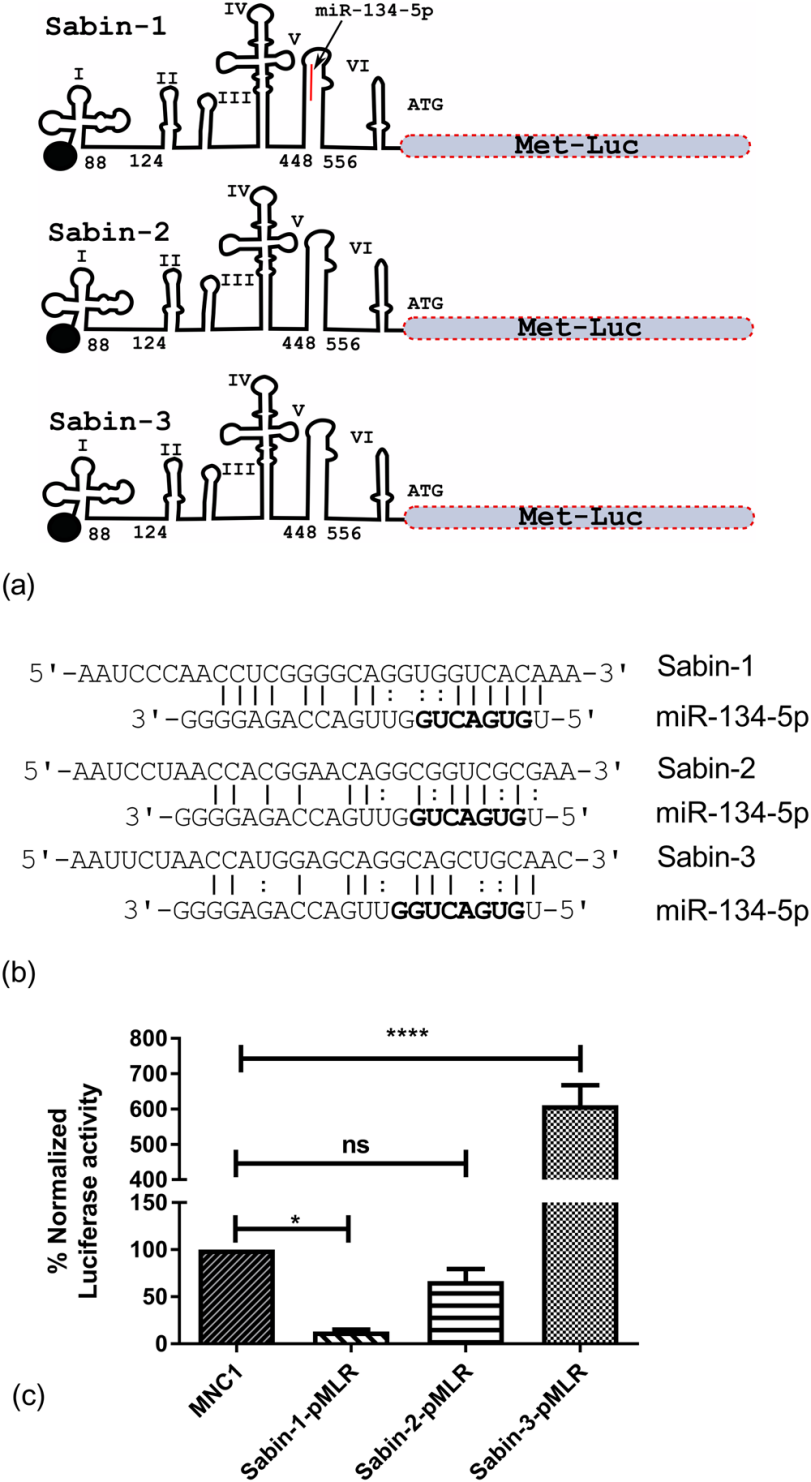
Reporter assays validate miR-134 inhibition of Sabin-1 but not Sabin-2 or Sabin-3 translation. (**a**) Design of reporter constructs for Sabin-1, Sabin-2, and Sabin-3 plasmids. Binding site for miR-134 is indicated for Sabin-1 in red (loop V). (**b**) Alignments of miR-134 and Sabin-1, Sabin-2 and Sabin-3 IRES are shown. Solid lines indicate Watson-Crick base pairs while colon symbols represent non-Watson-Crick pairs. Bold residues indicate miR-134 seed site. (**c**) Constructs above were co-transfected with control/miR-134 mimic and pSEAP2 Control (transfection control plasmid) into HEp-2 cells using Lipofectamine 2000. Luciferase expression was determined 48 h post transfection and normalized to SEAP expression. Control mimic (MNC1) values represent 100%. Error bars represent ± SEM from two independent experiments with triplicate replicates each. *p < 0.05, ****p < 0.001.

To test if miR-134 could bind to and suppress Sabin-1 IRES mediated translation, we cloned the 5′-UTRs of Sabin-1, Sabin-2 and Sabin-3 upstream of a *Metridia* luciferase reporter plasmid, pMetLucReporter Basic (pMLR) to generate Sabin-1-pMLR, Sabin-2-pMLR and Sabin-3-pMLR constructs, respectively (Fig. 3a). These were sequence validated and contained miR-134 binding region as identified computationally (Fig. 3b). Endotoxin-free, Sabin-1/Sabin-2/Sabin-3 pMLR plasmids were co-transfected into HEp-2 cells aseptically along with either a miR-134 mimic or a mimic negative control (MNC1), a miRNA mimic that targets *C. elegans* miR-67 (*cel-miR-67)* and has been shown to not alter human miRNA activity^28,56^. A plasmid that expresses secreted alkaline phosphatase (SEAP) under the control of a CMV promoter was used as a transfection control as shown previously^28,56^. Transfection of MNC1 did not inhibit luciferase expression from Sabin-1/Sabin-2/Sabin-3 pMLR plasmids supporting the non-specificity of MNC1^28^. SEAP expressions across all transfections were comparable. MiR-134 mimic transfections substantially reduced luciferase expression in Sabin-1-pMLR (Fig. 3c), but did not repress Sabin-2- or Sabin-3 IRES driven luciferase activity (Fig. 3c). These data support our premise that miR-134 can inhibit PV replication by interaction with the IRES in the Sabin-1 5′UTR.

**Figure 4:**
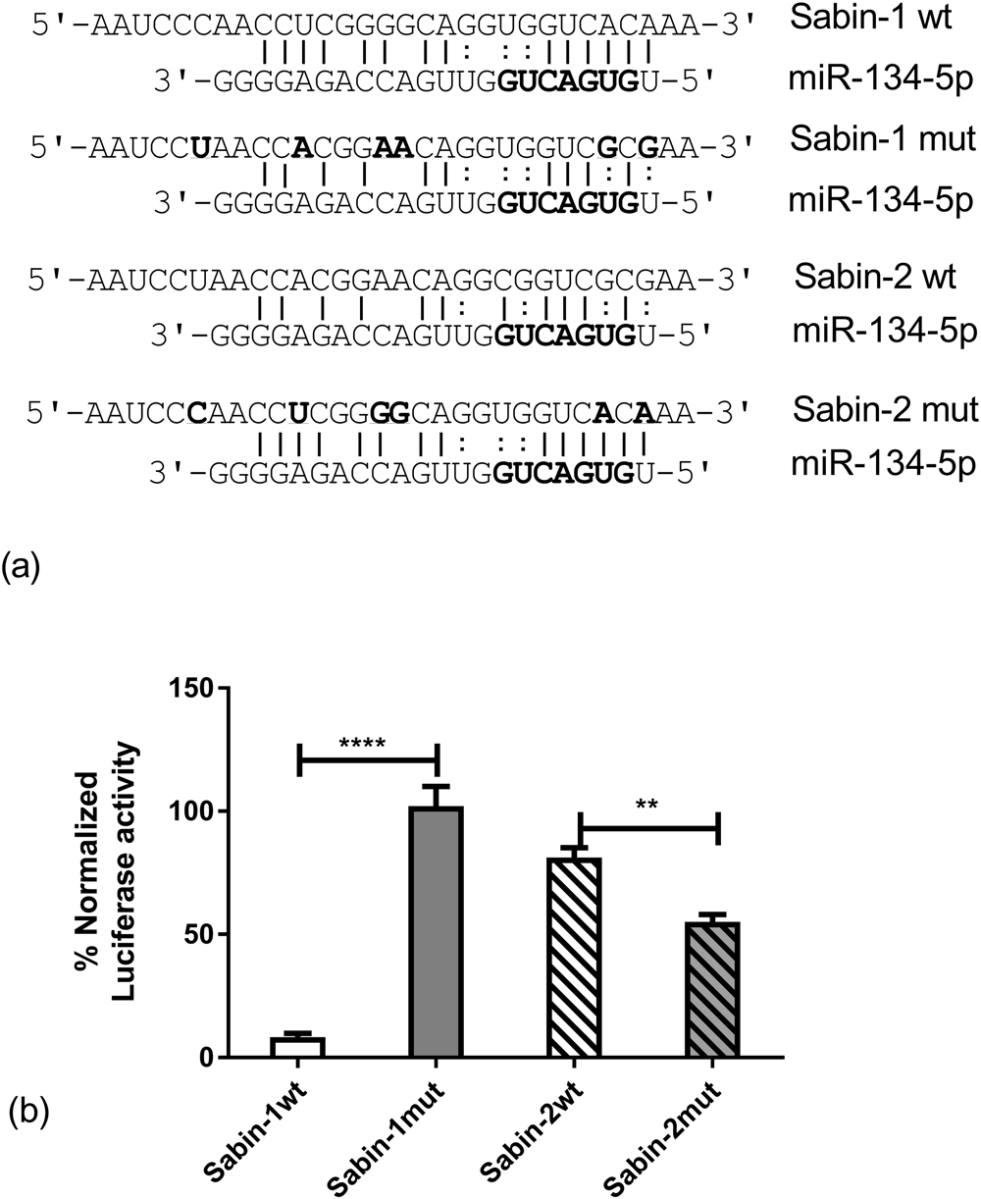
Site directed mutagenesis validates miR-134 targeting of the Sabin-1 IRES. (**a**) Alignments of wild type and mutated Sabin-1 and Sabin-2 sequences are shown aligned to miR-134 sequence. Solid lines indicate Watson-Crick base pairs while colon symbols represent non-Watson-Crick pairs. Bold residues in Sabin-1/2 sequences indicate residues that were mutated. **(b)** Sequence verified endotoxin free wild type (wt)/mutant (mut) plasmids were co-transfected into HEp-2 cells with control/miR-134 mimic and pSEAP2C control plasmid. Luciferase expression was assayed at 48 h post transfection. Mimic control (MNC1) values are set to 100%. Error bars represent ± SEM from two independent experiments in quadruplicate. *p < 0.05, **p < 0.01, ***p < 0.001 ****p < 0.0001. ns = non-significant.

To validate the specificity of inhibition of Sabin-1, but not Sabin-2 UTR driven luciferase expression by miR-134, we used directed mutagenesis of Sabin-1-pMLR and Sabin-2-pMLR plasmids. Mutant Sabin-1-pMLR plasmids contained a miR-134 complementarity sequence identical to wild type Sabin-2 (S1mut) while mutant Sabin-2-pMLR plasmids contain a miR-134 complementarity sequence identical to Sabin-1 (Fig. 4a). Thus mutant Sabin-1-pMLR constructs should be able to escape miR-134 inhibition while mutant Sabin-2 pMLR plasmids should be inhibited by miR-134. Equal amounts of sequence verified endotoxin-free wild type or mutant plasmids were co-transfected with MNC1/miR-134 mimics into HEp-2 cells as described. Plasmids encoding SEAP were transfected as a control in parallel. Data were normalized to control (MNC1) transfections and expressed as percentage activity relative to control. Expression of SEAP was equivalent across all transfections validating equivalent transfection efficiency. Transfection of the miR-134 mimic substantially inhibited wild type Sabin-1 driven luciferase activity. However, mutating the miR-134 binding site in Sabin-1-pMLR led to a noteworthy increase in luciferase activity (Fig. 4b). Conversely, the miR-134 transfection had little effect on Sabin-2 wild type IRES driven Luciferase activity. However, mutating the Sabin-2 miR-134 region to resemble Sabin-1 allowed for gain of miR-134 binding ability in Sabin-2 and repressed luciferase expression. These data establish that miR-134 can bind to and regulate Sabin-1 but not Sabin-2 IRES and regulate PV gene translation.

### Mir-134 binding degrades Sabin-1 IRES

**Figure 5:**
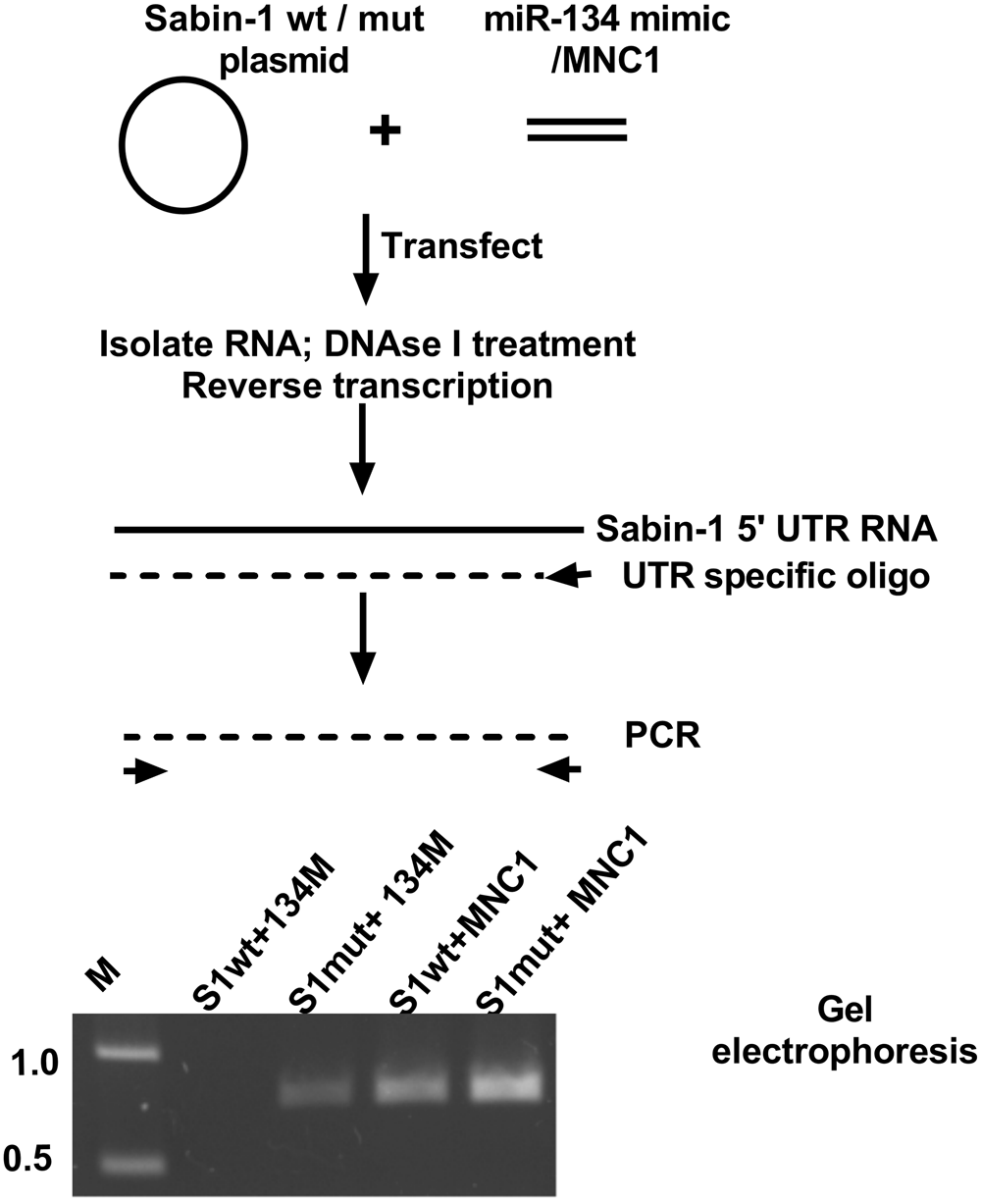
MiR-134 binding causes degradation of Sabin-1 IRES. HEp2c cells were co-transfected with endotoxin-free Sabin-1 wild type (S1wt) or mutant (S1 mut) IRES-luciferase and miR-134 mimic (134 M)/MNC1 (25 nM) for 24 h. Stability of Sabin-1 IRES was determined by Sabin-1 IRES specific PCR from equal amounts of cDNA from DNAse I treated total RNA. PCR products were resolved on 1% agarose gels containing 0.5 µg/ml Ethidium bromide in 1X TAE buffer at 3 V/cm. Image shows region of gel containing band of expected size representative of two independent experiments. Marker size is in kb.

We hypothesized two independent mechanisms of miR-134 inhibition of Sabin-1 UTR driven luciferase expression. In the first mechanism, binding of miR-134 does not alter stability of the IRES transcript but instead prevents IRES mediated translation of the PV-1 polyprotein. Alternatively, miR-134 could alter the stability of the PV-1 IRES and curtail IRES mediated translation of the PV-1 polyprotein completely. To determine if stability of the IRES transcript is altered by miR-134 binding, cells were co-transfected with Sabin-1 wild type/mutant plasmid and miR-134 mimics or control miRNAs as stated previously for 24 h. Total RNA was isolated and treated with DNAse I and quantified with RNA specific fluorescence based sensitive Qubit assay. Equal amounts of total RNA from all treatments were reverse transcribed using a validated Sabin-1 IRES specific reverse oligo. First strand cDNA was amplified with Sabin-1 specific forward and reverse oligos using a high fidelity polymerase to prevent mispriming. Electrophoresis of the PCR products showed a lack of IRES amplification in cells co-transfected with Sabin-1 wild type plasmid and miR-134 mimic but not in case of the mutant plasmid or control miRNA transfected cells (Fig. 5). These data clearly support mechanism 1 where binding of miR-134 at the binding site causes degradation of the Sabin-1 IRES and thus ablates IRES driven translation. Additionally, sequence analysis indicated that miR-134 could also potentially bind and regulate PTBP1 and eIF4B but does not seem likely given the short period of miR-134 mimic exposure.

**Figure 6:**
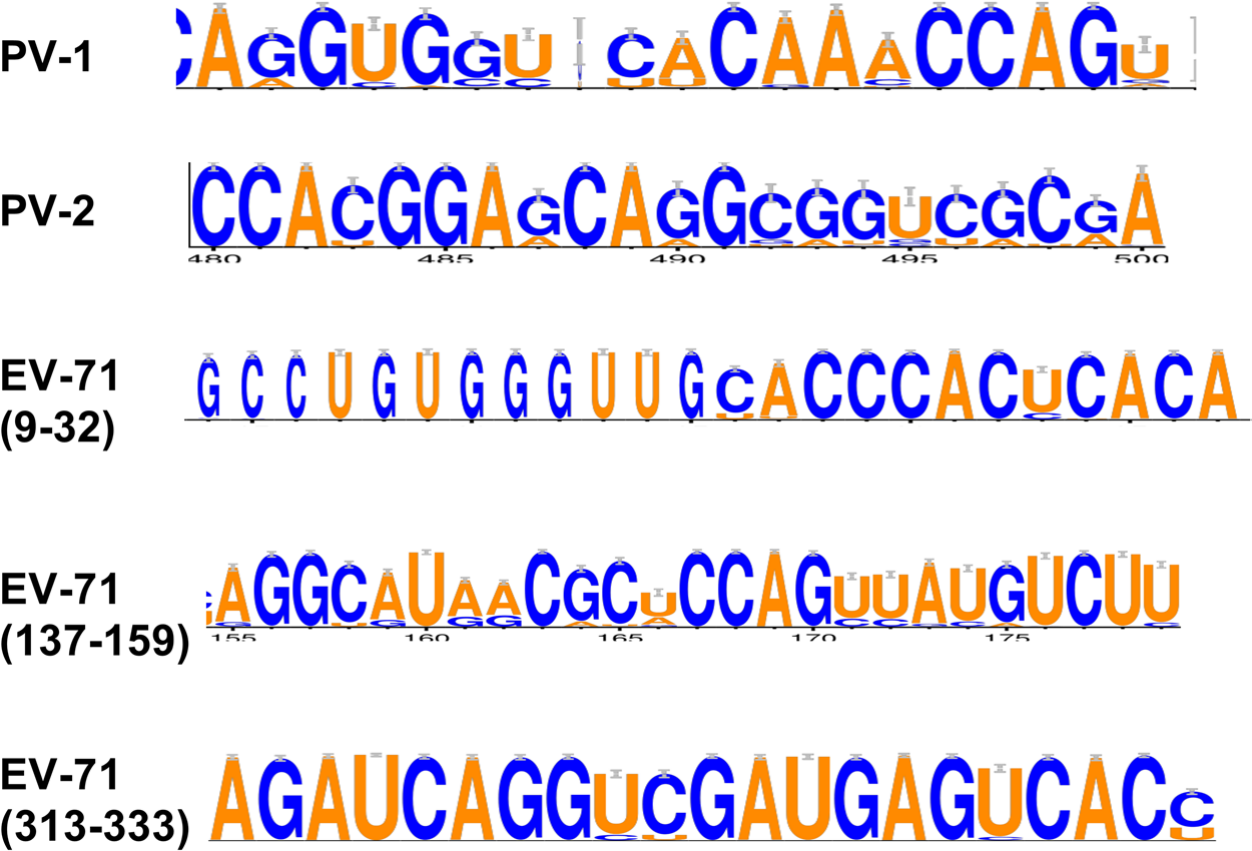
Sequence logos of miR-134 binding sites in PV-1and EV71 but not PV-2 IRES regions show extensive conservation. Genomes of wild type Sabin-1 (n = 218), Sabin-2 (n = 284) and EV71 (n = 615) sequences were aligned using CLUSTALW and conservation if any of putative miR-134 binding site was analyzed using WebLogo 3.0. The sequences represent consensus miR-134 binding site as predicted by computational algorithms in these regions of the PV-1, PV-2 and EV71. The height of each letter corresponds to degree of conservation in the dataset at the position examined.

Further, we investigated if this mechanism is restricted to the type strains used or is conserved among wild type clinical isolates. To determine this, wild type clinical sequences of PV-1, PV-2 and EV71, a related enterovirus were analyzed. Sequences were analyzed using CLUSTAL W. Sequence conservation at the putative miR-134 binding site across all sequences was plotted with WebLogos 3.0 using sequence logos (Fig. 6) where height of each residue is proportional to its conservation. Analysis clearly showed that miR-134 binding site was highly conserved in PV-1 but not in PV-2 sequences. Analysis of EV71 sequences identified three potential miR-134 binding sites in the EV71 IRES all of which were also highly conserved in wild type EV71 strains. These analyses clearly suggest that miR-134 may be able to bind and regulate the translation of circulating PV-1 and EV71 strains.

## Discussion

PV is nearly eliminated from the developed world but incomplete vaccine coverage and vaccine-derived PV hinder eradication. OPV has also increased risks of VDPV in the immune compromised individuals necessitating effective anti-PV therapeutics. Strategies that target viral components are effective but carry the risks of revertant or mutant viruses. Thus, host directed therapeutics that reduce viral burden can reduce the reemergence of wild type variants. Among the multiple therapeutic options under investigation, insights into the role of non-coding miRNAs at the host-PV interface can help identify pathways critical for viral replication and novel intervention strategies.

In previous studies, we showed that overexpression of six miRNAs markedly reduced PV replication in HEp-2 cells of which miR-555 was validated to regulate heteronuclear ribonucleoprotein C^28^. We also identified miR-134 as a key anti-PV miRNA and showed that it altered replication of Sabin-1, Sabin-2, and Sabin-3 post-transfection^28,30^. In these studies, we showed that a 48 h miR-134 mimic transfection inhibited replication of PV and EV71 in a virus and strain-independent manner by knockdown of RAN. Among other miR-134 regulated pathways, we showed regulation of RAN to be a major pathway affecting PV replication in a strain-independent manner. However, inducing miR-134 activity following infection repressed only Sabin-1, but not Sabin-2 or Sabin-3 replication (Fig. 1), suggesting an alternative mechanism of regulation. Changes in viral replication upon miRNA modulation were modest but consequential as has been shown with several other studies^35,36,57–60^. In this study, we show that miR-134 interacts with nts 472–492 in the domain V of Sabin-1 5′UTR. Sequence analysis identified perfect Watson-Crick complementarity between the miR-134 seed site and Sabin-1 5′-UTR. In case of Sabin-2 and Sabin-3, the miR-134 seed site complementarity contained both Watson-Crick and wobble base pairs reducing the predicted stability of the IRES: miRNA duplex.

Transfection of miR-134 mimic inhibited translation of Sabin-1 UTR driven Metridia luciferase in HEp-2 cells. In contrast, miR-134 did not inhibit translation of Sabin-2 or Sabin-3 UTR-driven reporters. We observed increased luciferase expression for Sabin-3 relative to Sabin-2 in miR-134 mimic transfected cells; the mechanisms involved are presently unknown and outside the scope of this study. These data show that miR-134 can bind to Sabin-1 UTR in a sequence-specific manner to inhibit PV translation. Further, substituting the Sabin-1 miR-134 binding site with Sabin-2 site relieved miR-134 suppression of Sabin-1 IRES driven luciferase. Conversely, Sabin-2 IRES driven luciferase expression was considerably reduced when the Sabin-1 miR-134 binding site was inserted into Sabin-2 plasmid. Analysis of other miR-134 binding sites in the PV genomes showed most to be conserved between strains (Supplementary Fig S1), and thus could not explain the selective inhibition of Sabin-1 by miR-134 observed previously (Fig. 1). Analysis of Sabin-1 IRES transcripts in cells transfected with wild type/mutant plasmids in the presence of miR-134 mimic/control miRNAs showed that miR-134 caused degradation of the Sabin-1 IRES. The Sabin-1 binding site is highly conserved in circulating Sabin-1 strains as well as in the related enterovirus EV71. This is the first report of a miRNA directly targeting the PV IRES leading to degradation of the IRES transcript and repression of IRES mediated polyprotein translation. These findings implicate a potential prophylactic/therapeutic role for miR-134 in ablating or reducing PV-1 shedding in OPV vaccinees.

miR-134 is expressed in multiple human tissues (pancreas, saliva and breast tissue, brain tissue^61^), as well as in the small intestine, the target tissues for PV replication. miR-134 expression is regulated by neuronal activity in response to multiple cellular and environmental cues^62^. In conjunction with other miRNAs specifically miR-34c-5p, miR-124-3p, miR-132-3p, and miR-212-3p, it has been shown that miR-134 can regulate proteins that regulate transcription upon synaptic activity^62^. In neuronal cells, miR-134 expression is regulated by Sirtuin-1 (SIRT1) and miR-134 regulates synaptic plasticity and memory^63^. MiR-134 also regulates formation of dendrites from neuronal stem cells (NSCs) via regulation of pumilio homolog 2 (Pum2)^64,65^. In a normal physiological state, miR-134 regulates the spinal volume in hippocampal neurons via LIM domain kinase 1 (Limk1)^66^. These findings show that multiple pathways are affected by miR-134 and suggest there may be other ways miR-134 may regulate PV replication.

Translation of the PV polyprotein is dependent on residues in the IRES in the 5′ UTR. PV and other enterovirus IRES sequences form a highly structured 5′UTR organized into 7 distinct domains^67^. This structure is highly conserved across serotypes and strains suggesting that it has an important role in viral polyprotein translation. Mutations or deletions in the different domains of the PV IRES are lethal^67^. Additionally, it was shown that pyrophosphate modified oligomers (PPMOs) targeting the domain 5 region of PV are able to inhibit multiple picornavirus species either by disrupting the IRES secondary structure and preventing interaction with the 40 S ribosomal subunit or by preventing binding of multiple IRES trans-activating factors (ITAFs), or by modifying the poly(RC) binding protein 2 (PCBP2)^68,69^, splicing factor SRP20/SRSF3^70^, EIF4B^71^, ribosomal protein 6 (RPS6)^72^, ribosomal protein 25 (RPS25)^73^, or polypyrimidine tract binding protein1 (PTBP1) which have been shown to be essential for PV replication. Targetscan analysis of miR-134 targets identifies PTBP1, and eIF4B as targets^74^. PTBP1 has been shown to interact with the base of domain V in cooperation with EIF4G^75^ and stimulates PV IRES activity^75^. Mutations at nt 472 which are associated with neuro-attenuation are also linked to decreased binding of PTB in neuroblastoma cells^75^. While it is possible that miR-134 overexpression reduces PTBP1 expression, and thus reduces IRES-mediated Luc translation for Sabin-1, it does not explain the lack of reduction for reporter expression for Sabin-2 and Sabin-3. It has also been shown that EIF4B binds to domain V in a PTB-independent but sequence-dependent manner and mutations in nt 482 and 529 of domain V reduce EIF4B binding and PV translation^71^. Since miR-134 interaction with domain V extends from nt 472–493, it is possible that it either reduces EIF4B levels, or prevents EIF4B binding leading to a loss in translation. These data suggest that miR-134 can inhibit PV genome replication in a strain-dependent manner via degradation of viral RNA by targeting the IRES.

## Materials and Methods

### Cell culture and viruses

HEp-2 cells were grown in high glucose DMEM (Hyclone) supplemented with 5% heat inactivated FBS (Hyclone). The PV strains were kindly provided by Dr. Steve Oberste (CDC, Atlanta) and viral titers were determined by plaque assays on HEp-2 cells and 50% cell culture infectious dose (CCID_50_)^76^.

### Transfection and cytotoxicity assays

RNA inference (RNAi) including miRNA mimics and inhibitors were reverse transfected into HEp-2 cells (8000/well) at a final concentration of 25 nM using Dharmafect-4 (DF4) per the manufacturer’s recommendation. Plates were further incubated at 37 °C/5% CO_2_ and 95% humidity before assaying for cytotoxicity, infection or RNA isolation. Cells were infected with PV strains, Sabin-1, Sabin-2 and Sabin-3, in 2% FBS for 24 h as described^77^. Cell supernatants were stored till further analysis. Cytotoxicity was measured 48 post-transfection using CytoxGlo assays (Promega) following manufacturer’s recommendations and siTOX positive control.

### Plaque assays

Plaque assays on HEp-2 cells were used to determine viral titers^78^. Briefly, 1.2 × 10^6^ cells/well were plated in six-well plates overnight. Monolayers were washed twice with MEM containing 2% FBS followed by an hour long incubation with 10-fold dilutions of the stock virus/cell supernatants. Inoculum was removed and plates were overlaid with nutrient agarose gel (0.9% agarose + 2X MEM supplemented with 4% FBS in a 1:1 ratio). Assays were incubated at 37 °C/5% CO_2_ and 95% humidity for 3 days followed by fixation in 10% formaldehyde and crystal violet counterstaining.

### RNA Extraction and RT-PCR

Total RNA was extracted using RNAzol RT (MRC gene) per the manufacturer protocol. Concentrations were determined using Nanodrop 1000 (ThermoFisher Scientific) spectrophotometry followed by first strand cDNA synthesis using 500 ng RNA across all samples and treatments using miRNA 1^st^ strand cDNA synthesis kit (Agilent Technologies). Primer sequences used for qPCR were as follows: miR-134-5p (5′-TGTGACTGGTTGACCA GAGGGG-3′ and proprietary adaptor reverse primer).

### Hypochromicity assays

Oligonucleotides corresponding to nt 473–493 for Sabin-1 (S1F 5′-ACCTCGGGGCAGGTGGTCACAAA-3′), nt 472–493 of Sabin-2 (S2F 5′-AACCACGGAACAGGCGGTCGCG-3′) and nt 475–498 of Sabin-3 (S3F-AACCATGGAGCAGGCAGCTGCAAC-3′) miR-134-5p (5′- TGTGACTGGTTGACCA GAGGGG -3′) were obtained commercially (Integrated DNA technologies). Working stocks (10μM) of S1F/S2F/S3F alone or mixed with miR-134 in equimolar amounts were heated to 65 °C for 5 minutes and then snap frozen on ice. Reactions were allowed to equilibrate to room temperature to allow hybridization and absorbance in the UV range was measured using a Tecan Safire × 2 spectrofluorimeter (n = 30).

### Cloning of Sabin-1, Sabin-2, and Sabin-3 UTRs for reporter assays

The 5′UTRs of Sabin-1 (Accession no. AY184219), Sabin-2 (AY184220) and Sabin-3 (AY184221) were cloned into pMetLuc Reporter (pMLR) plasmid (Clontech, Takara Biosciences, USA) to obtain S1-pMLR, S2-pMLR and S3-pMLR plasmids. Briefly, strain specific UTRs were amplified using common forward oligo SAB123UTRF (5′-GCGCG**AGATCT**TTAAAACAGCTCTGGGGTTG-3′) containing a BglII restriction site and Sab1GCUTRev (5′-GCGCG**CTGCAG**TATGATACAATTGTCTGATT-3′) or Sab2GCUTRev (5′-GCGCGCTGCAGTGTAGTATTGTTGTTTTATCC-3′) or Sab3GCUTRev (5′ GCGCGCTGCAGTGTGACACTGAAATCCTGTCTTC-3′) containing a PstI site, Sabin-1, -2 and -3 viral genomic DNA in pBR322 (kindly provided by Dr. Vincent Racaniello) as template and Q5 Taq DNA Polymerase under the following conditions: Initial denaturation 98 °C/30 seconds, 30 cycles of 98 °C/10 s and annealing extension at 72 °C/30 s and final extension at 72 °C for 7 minutes. PCR products were electrophoresed on a 1.2% Agarose gel in 1X TBE, expected sized bands (~750 bp) were cut out and eluted using a Wizard gel extraction kit (Promega). Eluted insert DNA and pMLR plasmid were digested overnight with BglII and PstI in NEbuffer 3.1 (New England Biolabs) per the manufacturer’s recommendations. Vector plasmid (pMLR) was dephosphorylated using Antarctic Shrimp phosphatase (New England Biolabs) and re-extracted using Wizard gel extraction kit (Promega). Dephosphorylated pMLR plasmid and Sabin-1, Sabin-2, Sabin-3 inserts were quantified using QuantiT-dsDNA assays (Invitrogen) and a Qubit flurometer, ligated in a 1:1 ratio with T4 DNA Ligase (New England Biolabs) at room temperature for 30 minutes and then transformed into JM109 competent cells (Promega). Transformants were plated on to LB agar plates containing 100 μg/ml Kanamycin and incubated overnight. Single isolated colonies were screened using colony PCR using above primers and recombinants were validated with restriction digestion and sequencing. All constructs were 100% identical to parental sequence. Transfection ready endotoxin-free DNA for all constructs, empty vector and transfection control plasmid pSEAP2Control were prepared using Qiagen maxiprep kit. A plasmid that expresses secreted alkaline phosphatase under the control of a CMV promoter (pSEAP2C/pS2C) was used as a transfection control. All recombinant plasmids generated in this study are available upon reasonable request.

### Site directed mutagenesis

Site directed mutagenesis of the parent S1-pMLR and S2-pMLR plasmids above was performed using Q5 site directed mutagenesis kit (New England Biolabs, USA) as per manufacturer’s protocol. Briefly, parent S1-pMLR and S2-pMLR plasmids were exponentially amplified using Q5 proof reading polymerase, common reverse primer (5′-tccgtggttaggattAGCCGCATTCAG-3′) and either S1pMLR-S2seed (5′-acaggcggtcgcgaaCCAGTGATTG-3′) or either S2pMLR-S1seed (5′-acaggcggtcacaaaCCAGTGACTGGC-3′) specific for binding to S1-MLR and S2-pMLR respectively in a 25 ul reaction. Reaction was treated with a kinase-ligase-Dpn1 mixture for sequential circularization, ligation and digestion of parent plasmid template(s). Treated reactions were transformed into high efficiency NEB 5-α and selected onto LB-Agar with kanamycin (50 μg/ml). Recombinants were screened for positive clones with colony PCR and sequenced to verify constructs.

### Luciferase Assays

Transfection-ready DNAs (empty pMLR vector, S1-pMLR, S2-pMLR, S3-pMLR and pS2C control expressing secreted alkaline phosphatase under the control of a CMV promoter were quantified using QuantiT dsDNA reagent in a Qubit fluorimeter. HEp-2c cells (10^5^ cells/well) were grown overnight at 37 °C/5% CO_2_ and 95% humidity in DMEM containing 5% FBS in Costar 48 well plates. Cells were co-transfected with 1μg of empty pMLR/S1-pMLR/S2-pMLR/S3-pMLR and 20 ng of pS2C along with miR-134-5p mimic or mimic negative control 1 (MNC1) (25 nM final concentration) using Lipofectamine 2000 as optimized previously^56^. Cell supernatants were collected at 24 h and 48 h post-transfection and Luciferase (Luc) and secreted alkaline phosphatase (SEAP) activities were measured using a Dual glow kit (Clontech). Expression of Luc was initially normalized to MNC1 followed by normalization to SEAP^56^.

### Analysis of IRES stability

Endotoxin free plasmid DNAs from Sabin-1 wild type (S1wt), miR-134 mutant (S1mut) were transfected into HEp2 cells along with miR-134 mimic/mimic negative control 1 (25 nM) for 24 h. Total RNA was extracted using RNAzol RT as per manufacturer’s instructions, quantified and then treated with RNAse free DNAse I (Ambion, Invitrogen, USA) at 37 °C/30 minutes followed by inactivation of the DNAse I with 10 mM EGTA at 65 °C for 10 minutes. DNAse treated RNA was precipitated overnight with 2.5 M LiCl and 2.5 volumes absolute ethanol. DNA free RNA was quantified using RNA specific high sensitivity Qubit RNA assays as per manufacturer’s protocol and equal amounts were used for reverse transcription with Protoscript II (New England Biolabs, USA) using oligo Sab-1UTR-Rev (5′-CTGCAGTATGATACAATTGTCTGATT3′). First strand cDNA was next amplified using Sab1-GC-UTRF (5′-GCGCGCGATCTTTAAAACAGCTCTGGGGTTG-3′) and Sab-1UTR Rev above in a 25 µL reaction containing 200 nM each oligo, 2X Q5 Hot Start master mix using the following conditions, initial denaturation 98 °C/30 seconds followed by 25 cycles of 98 °C/10 sec, annealing at 68 °C/30 second and amplification at 72 °C/30 seconds. No RT reactions were run in parallel to determine absence of DNA. PCR products were resolved on a 1% agarose gel in 1X TAE buffer and imaged on a ProteinSimple gel Imager.

### Computational analysis of miR-134 binding sites in PV-1 and EV71 genomes

Whole genome sequences of PV-1, PV-2 and EV71 were mined from NCBI. Sequences were aligned using Clustal W on a local machine and alignments were then analyzed for miR-134 binding sites using WebLogo 3.0^79^. List of accession numbers is provided as Supplemental Material 2.

### Statistical analysis

All data represent ± SEM of at least two/three independent replicates. Statistical analysis were done using one way or two way ANOVA with Tukey’s multiple comparison at 95% confidence level in GraphPad Prism 5.0. P values *<0.05, **<0.01, ***<0.001, ****<0.0001.

## Electronic supplementary material

Supplementary information

Supplementary data
