# Supplementary material for: MicroRNA-134 regulates poliovirus replication by IRES targeting
Source: Sci Rep. 2017 Oct 4;7:12664. doi: 10.1038/s41598-017-12860-z (PMC5627394; doi:10.1038/s41598-017-12860-z)
Supplement: Supplementary file 1 — Supplementary information [file 41598_2017_12860_MOESM1_ESM.pdf]

MicroRNA-134 regulates poliovirus replication by IRES targeting

Abhijeet A. Bakre[1], Byoung-Shik Shim[1‡] and Ralph A. Tripp[1#]

[1]University of Georgia, College of Veterinary Medicine, Department of Infectious Diseases, 30602

# Corresponding Author: Room 201, Animal Health Research Center, 111 Carlton Street, Athens GA 30602

ratripp@uga.edu

(O) 706-542-1557

‡Present address: Scripps Research Institute, Jupiter, Florida, USA.

Running title: miR-134 regulates poliovirus replication.

Keywords: Polio virus, miR-134, IRES

Abstract word count: 178

Text word count: 5127

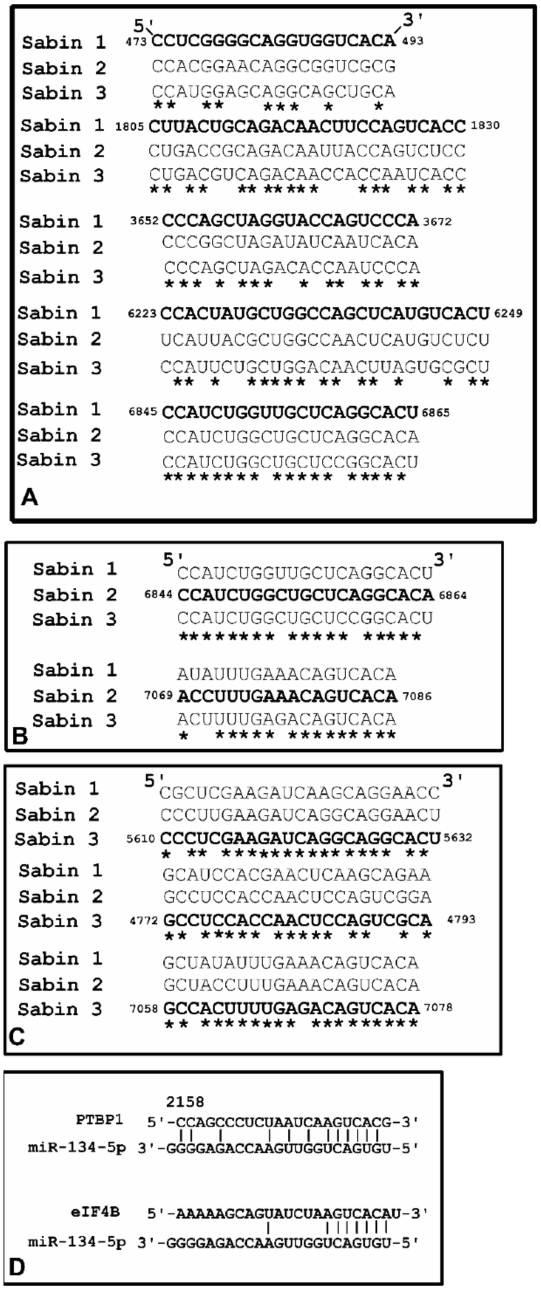

Supplementary Figure 1. CLUSTAL W alignments of miR-134 predicted binding sites in Sabin-1, Sabin-2 and Sabin-3 genomes and regulation of PTBP1 and EIF4B. Regions where miR-134 is predicted to bind as per ViTA across Sabin-1 (A), Sabin-2 (B) and Sabin-3 (C) are shown. Sabin-1, Sabin-2 and Sabin-3 sequence is highlighted in bold respectively. Asterisks below figure indicate conserved nucleotides across all three strains. (D) Alignment of miR-134 and PTBP1 and EIF4B are shown. Straight lines indicate Watson-Crick bonding.
